# Airway image analysis systems: a review of datasets, evaluation methodologies, and deep learning approaches

**DOI:** 10.3389/fdgth.2026.1962988

**Published:** 2026-10-22

**Authors:** Evropi Toulkeridou, Panagiota Kosmidou, Zinonas Antoniou, Marios S. Pattichis, Andreas S. Panayides

**Affiliations:** 1 VIDEOMICS, CYENS Center of Excellence, Nicosia, Cyprus; 2 Otolaryngologist Department, Mediterranean Hospital, Nicosia, Cyprus; 3 3AE Health LTD, R&D Department, Nicosia, Cyprus; 4 Department of Electrical and Computer Engineering, University of New Mexico, Albuquerque, NM, United States

**Keywords:** airway image analysis, airway segmentation, deep learning, explainable artificial intelligence, medical imaging, topology-aware segmentation, trachea segmentation, transformer networks

## Abstract

Airway image analysis systems play an increasingly important role in computer-assisted diagnosis, quantitative airway assessment, intervention planning, and respiratory disease management. However, the development of robust airway analysis methods remains challenging due to the complex geometry of airway structures, strong class imbalance, variability in imaging protocols, and the need to preserve anatomical connectivity across multiple spatial scales. Recent advances in topology-aware learning, transformer-based architectures, and foundation-model adaptation have substantially changed the landscape of airway image analysis, motivating an updated review focused specifically on airway-oriented systems. In this narrative review, we provide a comprehensive overview of the datasets, evaluation methodologies, and computational approaches that underpin modern airway image analysis systems. We discuss the imaging modalities and publicly available datasets commonly used for airway analysis and examine their strengths, limitations, and implications for algorithm development and benchmarking. Particular attention is given to evaluation methodologies, including overlap-based, surface-distance, and topology-aware metrics that assess both segmentation accuracy and structural correctness. We further review the evolution of computational approaches, covering convolutional neural networks, transformer-based architectures, hybrid methods, topology-aware and tubular-aware networks, foundation-model adaptations, and data-efficient learning strategies. Finally, we discuss current challenges related to dataset availability, annotation quality, model generalization, topology preservation, and clinical deployment, and outline future research directions toward robust, interpretable, and clinically deployable airway image analysis systems.

## 1 Introduction

Airway image analysis plays an increasingly important role in the diagnosis, monitoring, and treatment of respiratory and thoracic diseases (1, 2). Unlike general thoracic image analysis, airway analysis focuses on the extraction, characterization, and interpretation of highly connected tubular structures whose anatomical continuity is often critical for clinical decision-making. Advances in medical imaging and artificial intelligence have enabled the automated extraction of quantitative information from airway structures, supporting applications such as airway obstruction assessment, bronchoscopic navigation, tracheostomy planning, surgical guidance, and longitudinal disease monitoring (1–3). As imaging datasets continue to grow in size and complexity, automated airway analysis systems have become an active area of computer-assisted medicine. A fundamental task within airway image analysis is the accurate delineation of the trachea, bronchi, and surrounding thoracic structures. However, airway analysis presents challenges that distinguish it from conventional organ segmentation problems. The airway tree consists of thin, elongated, and highly branching tubular structures spanning multiple spatial scales. Small peripheral airways often occupy only a limited number of voxels, making them particularly susceptible to noise, partial-volume effects, and segmentation errors. Furthermore, substantial variability in patient anatomy, disease presentation, scanner hardware, and imaging protocols complicates algorithm development and limits model generalization across institutions.

Over the past decade, deep learning has transformed medical image analysis and has become the dominant paradigm for airway segmentation and related tasks (4, 5). Early studies relied primarily on convolutional neural networks (CNNs), particularly U-Net-based architectures, which demonstrated strong performance in delineating anatomical structures from volumetric medical images (6–11). More recently, transformer-based and hybrid architectures have introduced improved global context modeling, while topology-aware and tubular-aware approaches have been developed to preserve airway continuity and branching structure (12–22). At the same time, increasing attention has been devoted to foundation-model adaptation, semi-supervised learning, weak supervision, and active-learning strategies that address the scarcity of annotated airway datasets (23–33). Several reviews have summarized developments in general medical image segmentation and medical image analysis (4, 5, 34, 35). Importantly, Lsloum et al. recently published a 2026 systematic review specifically focused on machine-learning methods for fully automated airway segmentation on chest CT (36). That review synthesized 32 studies, including 28 deep-learning studies, and emphasized segmentation performance, topological completeness, external validation, and the predominantly retrospective nature of the available evidence. The present review is therefore positioned as complementary rather than as the first airway-specific review: it adopts a broader systems-oriented perspective linking airway datasets and benchmark resources, evaluation methodology, computational-model development, data-efficient learning, foundation-model adaptation, quantitative airway analysis, and the evidence required for clinical translation.

The objective of this review is to provide a comprehensive overview of the datasets, evaluation methodologies, computational models, and emerging research directions that underpin modern airway image analysis systems. This article is a narrative review informed by targeted searches of Scopus, IEEE Xplore, PubMed, and Google Scholar for literature on airway segmentation, thoracic image analysis, benchmarking, topology-aware learning, transformer and foundation-model methods, data-efficient training strategies, and clinically relevant airway-analysis studies. The scope of the present review differs from the recent systematic review by Lsloum et al. (36). Whereas that review systematically evaluates fully automated machine-learning methods for airway segmentation on chest CT, the present narrative review examines airway image analysis as a broader computational system. In addition to segmentation algorithms, it considers dedicated and related datasets, airway-specific evaluation and topology metrics, data-efficient and foundation-model approaches, quantitative airway analysis, and the progression from technical validation toward clinically meaningful applications. The two reviews should therefore be regarded as complementary rather than overlapping systematic syntheses. The main contributions of this review are threefold. First, we provide a focused analysis of dedicated airway benchmarks, thoracic imaging datasets with explicit airway or tracheal relevance, and related clinical cohorts, discussing their distinct roles in benchmarking, reproducibility, and model development (37–42). Second, we review and critically compare the principal methodological families used in airway image analysis, including convolutional, transformer-based, hybrid, topology-aware, tubular-aware, foundation-model, and data-efficient approaches. Third, we examine evaluation methodologies and translational evidence, distinguishing technical segmentation performance from external validation, prospective workflow evaluation, and demonstrated clinical utility.

Unlike existing surveys that primarily focus on general medical image segmentation, this review adopts an airway-centered perspective. Rather than treating airway segmentation as an isolated task, we consider its role within broader airway image analysis systems that support anatomical reconstruction, quantitative assessment, disease characterization, intervention planning, and clinical decision support. The remainder of this paper is organized as follows. Section 2 describes the literature search, study-selection approach, and reviews the evolution of research trends in airway image analysis systems. Section 3 discusses the imaging context and data sources that support airway analysis. Section 4 reviews publicly available datasets and benchmarking resources relevant to airway and thoracic imaging. Section 5 presents the evaluation methodologies commonly used in airway image analysis, including overlap-based, surface-based, and topology-aware metrics. Section 6 reviews the principal computational approaches used in airway image analysis, including CNN-based, transformer-based, hybrid, topology-aware, and data-efficient learning methods. Section 7 discusses current challenges, emerging research directions, explainability, and future opportunities. Finally, Section 8 concludes the paper.

## 2 Literature search and research trends

To identify relevant developments in airway image analysis, this narrative review was informed by targeted searches of Scopus, IEEE Xplore, PubMed, and Google Scholar. Search concepts included combinations of “airway segmentation,” “trachea segmentation,” “airway tree,” “thoracic image analysis,” “deep learning,” “topology,” “transformer,” “foundation model,” “semi-supervised,” and “weak supervision,” together with citation chaining from key airway benchmarks and reviews. The final literature search was updated on 20 July 2026. Figure 1 summarizes the literature identification process used in this review. Priority was given to peer-reviewed airway- or trachea-specific studies, primary benchmark publications, and methodological papers directly relevant to the review scope; additional general medical-imaging and clinical-context references were included where needed to explain the broader methodological or translational context. The review is not presented as a systematic or scoping review, and no PRISMA-style study-count denominator is claimed. Rather than providing a purely chronological catalogue, the review identifies the major technological and methodological trends that have shaped airway image analysis systems over the past decade.

**Figure 1:**
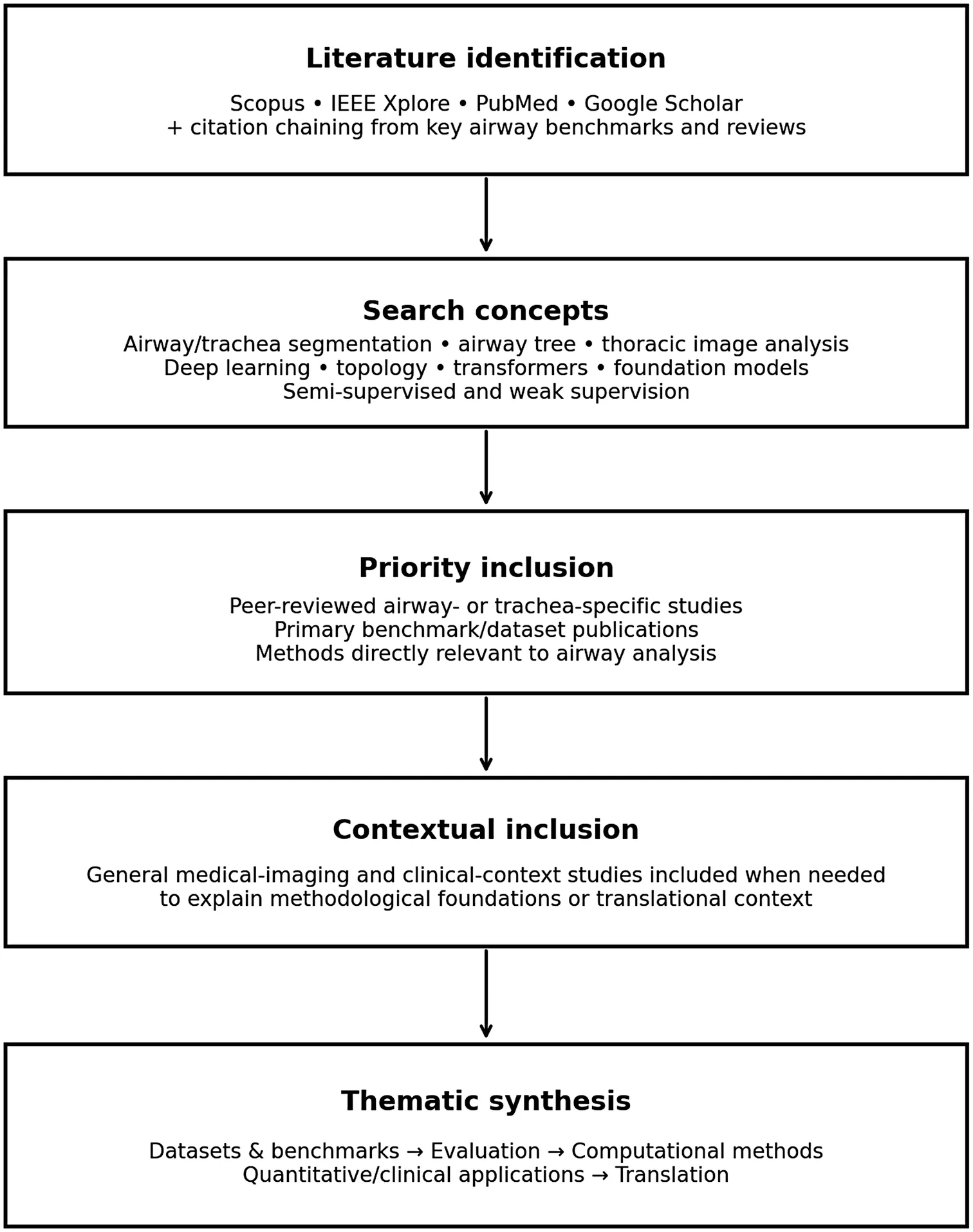
Literature identification and thematic inclusion process used in this narrative review. The diagram summarizes the targeted-search and source-prioritization strategy; it is not intended to represent a PRISMA-style systematic screening process, as formal screening counts were not prospectively recorded.

The evolution of airway image analysis has closely followed broader developments in medical image computing (1, 5, 34, 35). Early research was dominated by classical image-processing techniques and conventional machine-learning approaches that relied heavily on handcrafted features and rule-based segmentation pipelines (1, 2). These methods often struggled to cope with the complex geometry of the airway tree, particularly in the presence of anatomical variability, imaging noise, and pathological alterations. The emergence of deep learning fundamentally changed this landscape by enabling the direct extraction of hierarchical image representations from volumetric medical data.

The period between 2016 and 2020 was characterized by the widespread adoption of convolutional neural networks, particularly U-Net and its numerous variants (6–10, 43, 44). During this period, the primary objective was improving segmentation accuracy and achieving reliable delineation of major thoracic structures (4–6). Research largely focused on overlap-based performance measures such as Dice Similarity Coefficient and Intersection-over-Union, while most studies relied on relatively small datasets acquired from single institutions. Although these approaches achieved substantial improvements over traditional methods, challenges related to airway continuity, distal branch detection, and generalization remained largely unresolved (1, 26, 37).

Between 2020 and 2023, the field experienced a significant expansion driven by the availability of larger public datasets, benchmarking initiatives, and increasing computational capabilities (5, 39, 40). Hybrid architectures combining convolutional feature extraction with attention mechanisms emerged as an intermediate step toward more advanced models (13, 14, 16). At the same time, researchers began recognizing that segmentation accuracy alone was insufficient for airway analysis. The preservation of anatomical connectivity and branching structure became increasingly important, leading to growing interest in topology-aware learning, graph-based representations, and airway-specific evaluation methodologies (18, 20, 29, 31, 38).

Recent years have witnessed another major transition with the emergence of transformer-based architectures and foundation-model adaptations (12–15, 19, 22, 45). Models such as TransUNet, UNETR, Swin-UNet, and more recently MedSAM-derived frameworks have demonstrated the ability to capture long-range spatial dependencies while benefiting from large-scale pretraining (13–15, 29, 30, 32). These developments have shifted the focus of airway image analysis from isolated segmentation tasks toward more comprehensive systems capable of supporting quantitative airway assessment, disease characterization, intervention planning, and clinical decision support (1, 35).

The literature also reveals an important change in evaluation philosophy (5, 38). Earlier studies primarily emphasized volumetric overlap metrics, whereas recent research increasingly incorporates topology-aware measures, branch detection rates, centerline analysis, and anatomical completeness (18, 20, 38). This shift reflects a growing recognition that clinically useful airway analysis systems must preserve structural correctness in addition to achieving high numerical accuracy. Another notable trend is the increasing emphasis on data efficiency and model robustness. Semi-supervised learning, weak supervision, self-supervised pretraining, and domain-adaptation strategies have become active research areas as investigators seek to overcome the limited availability of expert airway annotations and improve cross-institutional generalization (23, 26–28). These approaches are particularly relevant for airway analysis, where the manual annotation of distal branches remains both labor-intensive and highly specialized.

Figure 2 summarizes research activity in airway image analysis from 2021 to 2026. The publication profile shows overall growth in activity with year-to-year variation, while the diversification of methodological approaches illustrates a broader transition from segmentation-focused studies toward more comprehensive airway image analysis systems. Collectively, these trends suggest that future progress will depend not only on improved segmentation accuracy but also on topology preservation, robust generalization, foundation-model adaptation, and clinically meaningful validation (18, 20, 21, 29, 30, 35). To further characterize the evolution of the field, the Scopus records used for the descriptive trend figure were grouped into broad thematic categories based on their primary methodological focus. The resulting distribution illustrates increasing representation of topology-aware learning, transformer-based architectures, foundation models, and clinically oriented airway analysis. The 2026 value is explicitly treated as a partial-year count rather than compared as a complete calendar year.

**Figure 2:**
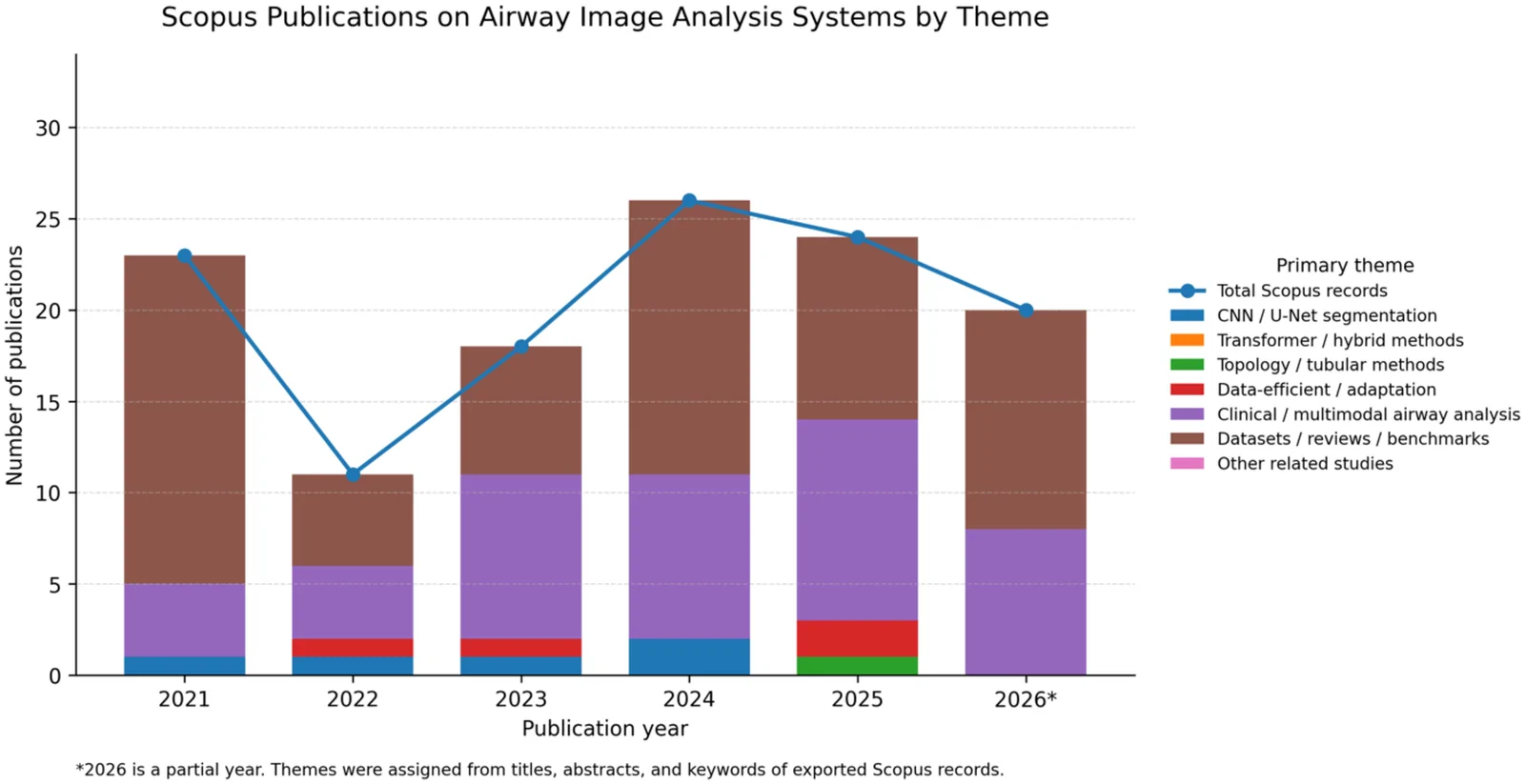
Scopus publication trends in airway image analysis systems from 2021 to 2026, grouped by primary thematic focus. The 2026 count represents a partial year. Themes were assigned from titles, abstracts, and keywords of the exported Scopus records.

## 3 Imaging context and data sources

Airway image analysis systems rely primarily on medical imaging data to characterize airway anatomy, detect pathological changes, and support clinical decision-making. Unlike many other anatomical structures, the airway tree consists of thin, elongated, and highly branching tubular structures that extend across multiple spatial scales, from the trachea to distal bronchi. These characteristics introduce unique challenges for image acquisition, segmentation, quantification, and visualization. Consequently, the choice of imaging modality, image quality, and dataset characteristics plays a fundamental role in determining the performance and clinical utility of airway image analysis systems.

Modern airway image analysis applications extend beyond simple anatomical delineation and increasingly support quantitative assessment, surgical planning, disease monitoring, and computer-assisted intervention (1–3). As a result, understanding the strengths and limitations of available imaging modalities and the specific challenges associated with airway anatomy is essential for the development of robust computational methods.

A. Imaging modalities

Medical image segmentation of thoracic anatomy relies primarily on imaging modalities capable of differentiating air-filled structures from surrounding cartilage, soft tissues, and vascular anatomy. For airway analysis, imaging systems must provide sufficient spatial resolution to accurately capture lumen boundaries, airway branching patterns, and relationships with adjacent anatomical structures.

1) Computed tomography (CT)

Computed Tomography (CT) remains the dominant modality for airway image analysis because of its high spatial resolution and ability to provide detailed volumetric representations of thoracic anatomy (1, 2, 37, 46, 47). The trachea and bronchi appear as low-attenuation air-filled structures surrounded by higher-density tissues, facilitating airway extraction and reconstruction. Modern multi-detector CT and high-resolution CT generate near-isotropic volumetric data that support three-dimensional visualization, morphometric analysis, and quantitative airway assessment. Dedicated airway benchmarks such as EXACT’09 and ATM’22 are CT-based (37, 38), and the majority of airway-specific deep-learning methods reviewed in this paper have consequently been developed and evaluated on CT data (16–22, 48–59).

2) Magnetic resonance imaging (MRI)

Magnetic Resonance Imaging (MRI) provides soft-tissue contrast without ionizing radiation and can complement CT in selected airway applications (60). Historically, MRI has been less frequently used for airway visualization because the low proton density of the lung and rapid signal decay at air–tissue interfaces reduce signal intensity. Developments in ultra-short echo-time (UTE) and zero-echo-time (ZTE) imaging improve pulmonary and airway visualization and may be particularly useful when repeated radiation-free imaging is desirable (60). Nevertheless, spatial resolution and acquisition constraints mean that MRI-based airway segmentation remains much less represented than CT-based analysis in the current evidence base. These modality differences also have implications for computational analysis. Compared with CT, the lower pulmonary signal, lower spatial resolution, and greater sensitivity to acquisition-related artifacts in MRI can affect preprocessing, boundary definition, distal-airway visibility, and the transferability of segmentation models developed primarily on CT. Although UTE and ZTE sequences can partially overcome the rapid signal decay at air–tissue interfaces, these techniques require specialized acquisition capabilities and are not universally available on routine clinical MRI systems. Consequently, results obtained using UTE/ZTE MRI may not generalize directly to conventional clinical MRI, and CT- and MRI-based airway segmentation methods should be validated within their respective imaging contexts.

B. Airway anatomy and image characteristics

The anatomical properties of the airway tree introduce several challenges that distinguish airway image analysis from conventional organ segmentation (18, 20–22). Unlike compact anatomical structures such as the liver or heart, the airway system consists of a connected hierarchy of tubular branches with rapidly decreasing diameters. Distal airways often occupy only a few voxels in medical images, making them particularly vulnerable to noise, partial-volume effects, and segmentation errors. The airway lumen is characterized by strong class imbalance, as airway voxels typically represent only a small fraction of the total image volume. Furthermore, airway visibility varies considerably across imaging protocols, scanner manufacturers, reconstruction parameters, respiratory phases, and patient populations. Disease-related changes such as airway narrowing, obstruction, inflammation, tumors, or mucus accumulation further complicate image interpretation and algorithm development. These characteristics explain why airway image analysis frequently requires specialized computational approaches that incorporate anatomical priors, connectivity constraints, topology preservation, and tubular structure modeling (18, 20–22).

C. Challenges associated with airway imaging

Several challenges continue to limit the performance and generalizability of airway image analysis systems. One of the most significant issues is the preservation of airway topology (18, 20–22). Small discontinuities in segmentation masks may lead to the loss of entire distal branches, resulting in anatomically implausible airway trees despite apparently high overlap metrics. Domain shift represents another important challenge. Variations in scanner hardware, acquisition protocols, reconstruction algorithms, and patient populations can significantly affect image appearance and model performance. As a result, models trained on a single dataset often experience performance degradation when applied to data acquired at different institutions. Annotation quality also remains a limiting factor. Manual airway labeling is time-consuming and technically challenging, particularly for distal bronchi (20, 23, 26, 37). Differences in annotation protocols and inter-observer variability can introduce inconsistencies that affect both model training and benchmarking. These limitations have motivated increasing interest in topology-aware learning, weakly supervised methods, domain adaptation techniques, and robust evaluation frameworks, all of which are discussed in later sections of this review (18, 20–23, 26–28).

D. Beyond imaging: future directions for integrated airway analysis

Although contemporary airway image analysis systems are primarily image-based, future clinical decision-support systems are expected to integrate image-derived biomarkers with complementary sources of information, including electronic health records, physiological measurements, longitudinal outcome data, and molecular biomarkers (35). Such multimodal integration may enable more comprehensive characterization of airway disease, support personalized treatment planning, and improve clinical decision-making. In the European context, future development and validation of such multimodal systems will also be shaped by the European Health Data Space (EHDS), which establishes a framework for secure access to and secondary use of electronic health data for purposes including research and innovation (61). However, the primary focus of current research remains the accurate extraction, representation, and analysis of airway anatomy from medical images. Consequently, the remainder of this review focuses on the datasets, evaluation methodologies, computational models, and clinical applications that underpin modern airway image analysis systems.

## 4 Datasets and benchmark resources

The performance and generalizability of airway image analysis systems depend heavily on the availability of high-quality annotated datasets (20, 37, 39, 40). Unlike segmentation tasks involving large anatomical structures such as the liver, heart, or kidneys, airway analysis requires accurate delineation of thin and highly branching tubular structures that are difficult and time-consuming to annotate. Consequently, the development of airway-specific datasets has lagged behind many other areas of medical image analysis (20, 26, 37, 38, 40). This limitation remains one of the most significant barriers to reproducible benchmarking, robust model development, and clinical translation (38–40).

Most airway image analysis studies rely on Computed Tomography (CT) datasets because CT provides sufficient spatial resolution to capture both central and peripheral airways (20, 37, 39, 40). However, relatively few publicly available datasets contain comprehensive airway annotations (20, 26, 37). As a result, many studies either use small private datasets or repurpose larger thoracic imaging resources originally developed for other clinical objectives. This situation has motivated increasing interest in standardized benchmark datasets and community challenges that facilitate objective comparison of competing methodologies (37, 39, 40). A summary of the most representative datasets is provided in Table 1.

**Table 1:** Representative datasets and benchmark resources used in airway image analysis research.

Dataset	Primary Structures	Modality	Cases	Main Use
EXACT’09 (37)	Pulmonary airway tree	CT	40 (20 training+20 test)	Classical airway-extraction benchmark
ATM’22 (38)	Detailed pulmonary airway tree	CT	500 (300/50/150)	Large multi-site/multi-domain airway benchmark
AeroPath (39)	Trachea and bronchi in challenging pathology	CTA	27	Pathology-focused airway benchmark
AirRC (72)	Airway lumen and wall; pulmonary arteries and veins	CT	254	Public multi-structure pulmonary segmentation resource based on LUNA16
SegTHOR (40)	Trachea, heart, aorta, esophagus	CT	60	Thoracic OAR/central trachea segmentation
StructSeg thoracic task (62)	Six thoracic OARs including trachea	CT	50 training cases	Multi-organ thoracic segmentation
LIDC-IDRI (41)	Lung nodules; no native complete airway-tree labels	CT	1,018	Thoracic source data/secondary airway use
COPDGene (42)	Thoracic CT and COPD phenotyping	CT	>10,000 participants	Airway morphology and disease phenotyping
KOLD (63)	COPD/asthma/obstructive disease cohort	CT	477 participants	Clinical airway disease analysis

A. Airway-specific datasets

Several resources serve as dedicated benchmarks for airway segmentation. EXACT’09 remains an important classical benchmark and contains 40 chest CT examinations divided into 20 training and 20 test cases (37). It established a standardized framework for comparing airway-tree extraction across heterogeneous scans. More recently, the Multi-site, Multi-domain Airway Tree Modeling challenge (ATM’22) substantially expanded benchmark scale and diversity, comprising 500 CT examinations with 300 training, 50 validation, and 150 test cases (38). ATM’22 is therefore a central contemporary benchmark for detailed airway segmentation and topological completeness. AeroPath complements these resources with 27 CTA examinations containing tracheal and bronchial annotations in anatomically and pathologically challenging cases (39). In contrast, LOLA11 is principally a lung/lobe segmentation challenge and VESSEL12 is a pulmonary vessel-segmentation challenge; neither is therefore presented here as a dedicated airway benchmark.

The differences among EXACT’09, ATM’22, and AeroPath are important for interpretation. EXACT’09 provides a long-standing extraction benchmark, ATM’22 adds substantially greater scale and multi-site/multi-domain diversity, and AeroPath emphasizes challenging pathology (37–39). These resources should not be treated as interchangeable because annotation extent, pathology, acquisition diversity, and evaluation criteria differ. Their complementary characteristics nevertheless provide a more appropriate basis for airway-specific benchmarking than datasets whose primary targets are lungs, lobes, vessels, or unrelated organs.

B. Thoracic and Multi-Organ Datasets

Because dedicated airway datasets remain limited, broader thoracic datasets can still be relevant provided that their annotation scope is stated accurately. SegTHOR contains 60 thoracic CT examinations with annotations of the trachea, heart, aorta, and esophagus and is therefore useful for central tracheal segmentation, but it does not provide complete bronchial-tree ground truth (40). The thoracic task of StructSeg contains 50 training CT cases and six thoracic organs at risk including the trachea (62). LIDC-IDRI contains 1,018 thoracic CT examinations centered on lung-nodule annotation and does not natively constitute an airway-tree segmentation benchmark, although its scans may be reused in secondary airway studies (41). COPDGene is a large COPD cohort with more than 10,000 participants and inspiratory/expiratory CT imaging, making it relevant to quantitative airway morphology rather than standardized airway segmentation benchmarking (42). The KOLD cohort contains 477 participants with COPD, asthma, or other obstructive lung disease and similarly represents a disease cohort rather than an airway segmentation challenge (63).

Large multi-organ datasets remain valuable for pretraining, transfer learning, and general segmentation-method development, but performance on such datasets is not evidence of airway-specific performance (5). This distinction is particularly important when benchmark tables are used to support conclusions about airway topology or distal-branch recovery. Generic abdominal multi-organ resources are likewise not listed as airway benchmarks unless they provide explicit thoracic or airway annotations.

The secondary use of thoracic datasets acquired for other clinical purposes also requires careful consideration of pathology. Pulmonary nodules, tumors, airway obstruction, mucus, atelectatic changes, or anatomical distortion may alter local airway appearance and potentially introduce bias during training or evaluation. Such abnormalities should not simply be removed, because they may represent clinically relevant conditions that airway-analysis systems are expected to handle. Instead, studies using repurposed datasets should characterize the included pathology, provide appropriate airway-specific reference annotations, and, where possible, report pathology-stratified performance to determine whether algorithms remain robust in the presence of adjacent or airway-altering abnormalities.

C. Benchmarking and dataset challenges

The evolution of airway image analysis has been accompanied by increasing emphasis on standardized benchmarking. EXACT’09, ATM’22, and AeroPath provide complementary evaluation environments for airway extraction, multi-domain robustness, topology, and challenging pathology (37–39). SegTHOR and StructSeg provide useful tracheal or thoracic-organ annotations but should be interpreted as thoracic multi-organ resources rather than complete airway-tree benchmarks (40, 62). These initiatives improve reproducibility and comparison only when their intended target structures, case counts, splits, and metric definitions are reported accurately.

Despite these advances, significant challenges remain. Annotation variability continues to affect model development because airway labeling is inherently difficult, particularly for distal branches that may occupy only a few voxels (21, 22, 53, 55, 64–68). Differences in annotation protocols, observer expertise, and quality-control procedures can introduce inconsistencies across datasets and complicate cross-study comparisons. Both inter-observer and intra-observer variability are particularly relevant for small peripheral branches and ambiguous airway boundaries, where the definition of “ground truth” may itself be uncertain. Reference annotations should therefore be interpreted as expert-derived estimates rather than error-free representations of airway anatomy, and studies should report annotation and adjudication procedures whenever possible. Variations in scanner hardware, acquisition parameters, reconstruction algorithms, pathology, and patient populations can also create substantial domain shifts. Consequently, performance on a single internal test set is insufficient evidence of broad generalizability, and external validation should be reported separately from internal cross-validation (35, 36, 69–71).

D. Future directions in airway data resources

The literature consistently identifies dataset availability as a major limiting factor in the development of airway image analysis systems (23, 37, 39, 40, 72). Future progress will likely depend on the creation of larger multi-institutional datasets that combine diverse imaging protocols with standardized annotation procedures. Particular attention should be given to distal airway labeling, topology-aware annotations, longitudinal follow-up studies, and cross-site validation protocols.

In addition, future airway benchmarks should move beyond segmentation accuracy alone and incorporate evaluation of anatomical completeness, robustness, clinical utility, and generalization (18, 20–22, 38). The integration of imaging data with complementary information sources, including physiological measurements and clinical outcomes, may further facilitate the development of clinically relevant airway analysis systems and support the next generation of multimodal airway artificial intelligence.

Collectively, these datasets reveal an important imbalance within airway image analysis. While large multi-organ datasets now contain hundreds of cases, dedicated airway datasets remain relatively small and often focus on specific anatomical regions or disease cohorts. This disparity partly explains the growing interest in transfer learning, foundation-model adaptation, and data-efficient learning strategies.

## 5 Evaluation methodologies

The evaluation of airway image analysis systems presents challenges that differ substantially from those encountered in conventional compact-organ segmentation (1, 36, 73). Because the airway tree consists of thin, elongated, and highly branching structures spanning multiple scales, evaluation must consider not only volumetric overlap but also boundary accuracy, structural continuity, distal completeness, leakage, and topological correctness (18, 19, 37, 54–58). Metrics that perform well for large organs may fail to capture important errors in distal airways or branch connectivity. Modern airway evaluation should therefore combine complementary overlap, surface, airway-specific, and topology-aware measures.

A. Overlap-based metrics

Overlap-based metrics remain widely reported because they provide intuitive assessments of agreement between predicted and reference segmentations. Dice Similarity Coefficient (DSC) and Jaccard Index/Intersection-over-Union (IoU) are common, while sensitivity/recall and precision provide complementary voxel-wise information about missed airway voxels and over-segmentation. However, these measures can overestimate airway-tree quality because large proximal airways contribute disproportionately to global overlap, whereas loss of peripheral branches or small topological discontinuities may have only a modest effect. Voxel-wise accuracy and specificity can be especially misleading in this highly imbalanced task because the background class dominates the image volume (36–38).

B. Surface-based metrics

Surface-based metrics quantify geometric discrepancies between predicted and reference boundaries. The Average Symmetric Surface Distance (ASSD) evaluates mean bidirectional surface distance, whereas Hausdorff Distance (HD), often reported as HD95 to reduce sensitivity to extreme outliers, captures larger local deviations. These measures are particularly relevant when segmentation supports airway diameter, stenosis, wall, or other morphometric measurements (1, 37, 73). Nevertheless, good surface agreement does not by itself guarantee preservation of the branching tree, so surface metrics should be interpreted together with overlap and airway-specific structural measures.

C. Topology-aware metrics

Topology preservation represents one of the defining challenges of airway analysis (18–22, 73). Established airway benchmarking therefore incorporates branch- and centerline-based measures. Tree Length Detected (TD) quantifies the proportion of the reference airway centerline length recovered, while Branch Detected (BD) quantifies the proportion of reference branches successfully recovered. EXACT’09 also reports branch count, tree length, leakage count, leakage volume, and false-positive rate (37). Contemporary benchmarks such as ATM’22 continue to emphasize structural completeness and topological correctness (38).

Completeness should not be optimized in isolation. A method may recover additional distal branches while simultaneously introducing leakage into adjacent structures or anatomically implausible extensions. Thus, increases in BD or TD should be interpreted together with false-positive and leakage measures (37, 38). This motivates a distinction between topological completeness—how much of the true airway tree is recovered—and topological correctness—whether the recovered structure remains anatomically plausible. Centerline- and graph-based analyses can further characterize connectivity and branch-level errors that are poorly represented by voxel overlap (18, 19, 74).

D. Statistical and clinical evaluation

Quantitative segmentation metrics may be complemented by statistical analysis and expert assessment. Confidence intervals and appropriate comparative statistics improve reporting rigor, while Bland–Altman analysis is useful when evaluating agreement in derived quantitative measurements such as airway diameter or other morphometric parameters (75–79). Agreement should, however, be distinguished from reproducibility. Bland–Altman analysis characterizes bias and limits of agreement between measurements or methods, whereas test–retest analysis evaluates the stability of measurements across repeated acquisitions. Inter- and intra-observer analyses can additionally quantify variability associated with reference annotation or measurement. These forms of repeatability and reproducibility are particularly important for longitudinal applications, in which apparent changes in airway dimensions or morphology must be distinguished from measurement variability.

Expert review can identify discontinuities, leakage, missed stenotic regions, and implausible reconstructions that may not be fully captured by numerical scores. However, retrospective expert assessment should not be interpreted as evidence of clinical impact. Technical validation, external validation, prospective workflow evaluation, and demonstrated effects on clinical decisions or outcomes represent distinct levels of evidence (35, 36).

Table 2 summarizes the principal categories of evaluation metrics currently employed in airway image analysis systems and highlights their respective strengths and limitations.

**Table 2:** Evaluation methodologies commonly used in airway image analysis systems.

Metric	Description	Typical Range/Units
Dice Similarity Coefficient (DSC)	Volumetric overlap between prediction and reference.	(0, 1)
IoU/Jaccard	Intersection relative to union.	(0, 1)
Sensitivity/Recall	Fraction of reference airway voxels recovered.	(0, 1)
Precision	Fraction of predicted airway voxels that are correct.	(0, 1)
Average Symmetric Surface Distance (ASSD)	Mean bidirectional surface distance.	mm; lower is better
Hausdorff Distance/HD95	Maximum or high-percentile surface discrepancy.	mm; lower is better
Tree Length Detected (TD) (37, 38)	Fraction of reference airway centerline length recovered.	%; higher is better
Branch Detected (BD) (37, 38)	Fraction of reference airway branches recovered.	%; higher is better
Branch count/tree length (37)	Extent of recovered airway tree.	Count/length
False-positive rate (37)	Fraction of the total detected airway volume classified as incorrect relative to the reference.	%; lower is better
Leakage count/leakage volume (37)	Number and volume of leakage regions.	Count/volume
Connectivity/centerline-based measures	Connectivity and centerline-based assessment of structural correctness and tree continuity.	Method-dependent
Bland–Altman analysis (75–79)	Agreement of derived quantitative airway measurements.	Bias and limits of agreement

No single metric can fully characterize airway segmentation quality. Overlap-based metrics provide useful measures of global segmentation accuracy, surface-based metrics quantify geometric precision, and topology-aware metrics evaluate structural correctness. Because clinically useful airway analysis requires both accurate boundaries and preserved anatomical connectivity, future benchmarking efforts should report complementary metric sets that jointly assess volumetric, geometric, and topological performance. Such a strategy provides a more reliable assessment of model suitability for real-world airway applications (18, 20–22).

## 6 Computational approaches

The development of airway image analysis systems has been driven by continuous advances in deep learning methodologies (1, 4, 5, 34, 35). Early studies focused primarily on CNN-based segmentation architectures that demonstrated strong performance for anatomical delineation tasks. More recent work has expanded toward transformer-based models, topology-aware frameworks, data-efficient learning strategies, and clinically oriented analysis systems (13–15, 18, 20, 21, 29, 30, 35). This evolution reflects the increasing complexity of airway image analysis, where accurate segmentation alone is insufficient and algorithms must also preserve airway connectivity, generalize across institutions, and support clinically meaningful decision-making. Based on the trends identified in Section 2, the reviewed methods can be grouped into five major categories as shown in Table 3: CNN-based segmentation approaches, transformer and hybrid architectures, topology-aware methods, data-efficient learning strategies, and clinical airway analysis applications.

**Table 3:** Representative computational approaches used in airway image analysis systems.

Category/Method	Year	Core Innovation	Relevance to Airway Analysis
1. CNN-Based Architectures			
U-Net (6)	2015	Encoder–decoder with skip connections	General foundation widely adapted to airway segmentation
Attention U-Net (8)	2018	Attention-guided feature selection	General architecture; airway evidence requires airway-specific study
MultiResUNet (9)	2020	Multi-scale feature representation	General segmentation foundation
nnU-Net (11)	2021	Self-configuring segmentation framework	Strong general 3D baseline; airway performance must be cited separately
Tubule-Sensitive CNN (54)	2021	Tubular-structure-sensitive learning	Direct pulmonary airway/artery-vein evaluation
2. Transformer and Hybrid Architectures			
TransUNet (13)	2021	CNN+transformer context modeling	General medical segmentation foundation
UNETR (14)	2022	Transformer encoder for volumetric segmentation	General 3D medical segmentation foundation
Swin-Unet (15)	2022	Hierarchical shifted-window transformer	General medical segmentation foundation
Two-stage contextual transformer CNN (16)	2023	Contextual transformer+CNN extraction	Direct airway CT evaluation
Adversarial Transformer (17)	2023	Transformer-based airway segmentation repair	Direct airway structural correction
3. Foundation Models and Prompt-Based Segmentation			
Segment Anything (SAM) (29)	2023	Promptable general segmentation	General foundation model; not airway-specific
MedSAM (30)	2024	SAM adapted to medical images	General medical foundation model
Medical SAM Adapter (31)	2025	Parameter-efficient medical adaptation	General medical adaptation
MedSAM2 (32)	2025	3D medical image/video segmentation	Emerging general medical foundation model
SAM for Medical Images (33)	2023	Evaluation/adaptation of SAM in medical imaging	General medical evidence; airway topology requires direct validation
4. Topology-Aware and Tubular-Aware Methods			
Connectivity-Aware Pulmonary Airway Segmentation (18)	2024	Connectivity-aware optimization	Airway-specific; targets disconnected branches
Anatomy-Aware Multi-Class+Topology-Guided Learning (19)	2024	Anatomical decomposition+topology guidance	Airway-specific completeness/correctness
Topological Structure Enhancement (48)	2024	Multi-task topology enhancement	Airway-specific topology modeling
Skeleton-guided 3D CNN (22)	2025	Skeleton-guided tubular segmentation	Tubular structural guidance
TfeNet (20)	2026	Direction-aware convolution+tubular feature fusion	Airway-specific; evaluates tubular branch recovery
5. Data-Efficient Learning Approaches			
Dual Consistency Learning (24)	2024	Semi-supervised consistency learning	Airway-specific semi-supervised method
Human–AI Active Learning (25)	2024	Human-in-the-loop active learning	Airway-specific annotation efficiency
SA-Seg (23)	2025	Annotation-efficient airway segmentation	Airway-specific
Skeleton2Mask (21)	2025	Sparse skeleton supervision	Reduces dense airway annotation burden
Mean Teacher (26)	2021	Consistency learning with unlabeled data	General semi-supervised foundation; not airway-specific

A. CNN-based segmentation methods

Convolutional neural networks (CNNs) established the foundation of modern medical image segmentation and remain widely used for airway analysis (4, 6, 7). Their ability to efficiently capture local spatial patterns, combined with relatively modest computational requirements, has made them particularly attractive for airway and thoracic imaging applications. The introduction of U-Net by Ronneberger et al. (6) represented a major milestone in medical image segmentation through its encoder–decoder architecture and skip connections, which preserve spatial information across network layers, while V-Net adapted fully convolutional learning to volumetric data (6, 7). Attention U-Net, MultiResUNet, UNet 3+, and nnU-Net subsequently explored attention, multi-scale representation, full-scale skip connections, and automated configuration (8–11). These are important methodological foundations, but their original papers should not automatically be interpreted as airway-specific evidence. Airway-specific CNN studies include leak-aware, graph-refined, tubule-sensitive, coarse-to-fine, and connectivity-aware approaches (54–59, 65–68, 74, 80). CNN-based architectures remain highly competitive because of their computational efficiency, training stability, and ability to achieve strong performance on relatively small datasets (18, 55). However, their reliance on local receptive fields often limits their ability to capture long-range anatomical dependencies. In airway segmentation, this limitation may lead to fragmented airway trees, missing distal branches, or reduced structural consistency despite high overlap scores.

B. Transformer and hybrid architectures

Transformer-based models introduced self-attention mechanisms that exchange information across distant image regions and can model broader anatomical context (12–15). General architectures such as TransUNet, UNETR, and Swin-Unet provide relevant architectural foundations but are not themselves airway-specific evidence (13–15). Airway-specific transformer work includes the two-stage contextual transformer-based CNN for CT airway extraction and the adversarial transformer for repairing human airway segmentation (16, 17). For airway analysis, global contextual modeling is attractive because spatially separated branches remain related within the same anatomical tree. However, transformers can require greater memory and training data, and global attention does not inherently guarantee connectivity; structural benefit must therefore be demonstrated using airway-specific benchmarks and topology-sensitive metrics.

For airway image analysis, transformer-based approaches are particularly attractive because airway structures extend across large spatial distances while maintaining complex branching relationships. However, these benefits come at the cost of increased computational complexity, larger memory requirements, and greater dependence on training data volume (13–15).

C. Foundation models and prompt-based segmentation

The introduction of foundation models has opened a new research direction in medical image analysis by enabling knowledge transfer from large-scale pretraining to specialized applications (29–33). SAM demonstrated prompt-based segmentation across natural images, motivating medical adaptations because expert annotation is expensive (29). MedSAM, Medical SAM Adapter, MedSAM2, and related work subsequently adapted this paradigm to medical images (30–33). These models are relevant to airway analysis as potential tools for transfer learning, interactive correction, and annotation acceleration, but they should be distinguished from airway-specific segmentation algorithms.

A major milestone was the introduction of the Segment Anything Model (SAM) by Kirillov et al. (29), which demonstrated broad prompt-based generalization across a wide range of segmentation tasks through prompt-based image segmentation. Although SAM was originally developed using natural-image datasets, its strong representation capabilities motivated extensive efforts to adapt the framework to medical imaging applications. Subsequent studies showed that direct application of SAM to medical images often produced suboptimal results because of the substantial differences between natural-image content and medical imaging characteristics. Consequently, several medical adaptations were proposed to improve performance on clinical datasets.

Among these developments, MedSAM (30) represents one of the most influential adaptations of the SAM framework for medical image segmentation. By retraining and fine-tuning the model using large collections of medical images, MedSAM demonstrated improved robustness and generalization across multiple anatomical structures and imaging modalities. Additional developments, including Medical SAM Adapter (31), MedSAM2 (32), and other domain-specific variants (33), further explored efficient adaptation strategies, three-dimensional extensions, and improved prompt-guided segmentation capabilities.

For airway image analysis, foundation models offer potential advantages in transfer learning, interactive annotation, and human-in-the-loop dataset construction (29–33). Such capabilities may be valuable for distal-airway labeling and annotation refinement. However, current evidence does not establish that general SAM-derived systems preserve fine peripheral branches or connected airway topology better than specialized airway networks. Direct comparison on dedicated benchmarks such as ATM’22 and AeroPath, using BD, TD, leakage, and overlap measures, is required before claims of improved airway performance or generalization can be made (38, 39).

Nevertheless, the emergence of foundation models represents one of the most significant recent developments in medical image analysis. Their ability to leverage large-scale pretraining while adapting to specialized clinical tasks suggests that they may play an increasingly important role in future airway image analysis systems, particularly when combined with topology-aware learning, weak supervision, and data-efficient training strategies.

D. Topology-aware and tubular-aware methods

Preserving airway topology is one of the most distinctive challenges in airway image analysis (18–22, 74). A segmentation may achieve high Dice while containing disconnected branches, missing distal airways, or false extensions. Airway-specific research has therefore incorporated skeleton guidance, centerline supervision, graph representations, connectivity-aware objectives, anatomical decomposition, and topology-guided iterative learning (18, 19, 21, 22, 48, 58, 74). Examples include graph-refinement methods (74), skeleton-guided CNNs (22), and direction-aware and airway-repair frameworks (17, 20). More recently, tubular-specific architectures such as TfeNet (20) have introduced anisotropic feature extraction mechanisms that explicitly exploit the geometric characteristics of tubular anatomical structures. These methods represent a shift from generic architectural innovation toward designs motivated explicitly by bronchial-tree geometry and topology.

The growing popularity of these methods reflects a broader shift within airway image analysis from purely overlap-based optimization toward anatomically meaningful reconstruction (18, 20, 21, 38). Topology-aware methods have become particularly important for distal airway segmentation, bronchial-tree extraction, and applications where airway completeness directly influences clinical utility.

E. Data-efficient learning and adaptation

A persistent challenge in airway image analysis is the limited availability of densely annotated datasets. Manual labeling of airway trees is labor-intensive and often requires expert knowledge, making large-scale annotation efforts difficult to sustain. To address this limitation, increasing attention has been devoted to semi-supervised, weakly supervised, and domain-adaptation strategies. Semi-supervised approaches utilize consistency regularization, pseudo-labeling, and self-training mechanisms to exploit large quantities of unlabeled data (24, 26, 27). Weakly supervised and sparse-supervision methods reduce annotation requirements through scribbles, saliency-based labels, or skeleton annotations (21, 23, 28). These approaches can reduce dependence on exhaustive voxel-wise manual annotation, but they do not inherently eliminate observer-related variability or ground-truth uncertainty, because sparse labels, skeletons, pseudo-labels, and other supervision signals may themselves contain annotation uncertainty and require appropriate validation.

Recent studies have further explored active learning and other annotation-efficient strategies aimed at reducing expert labeling burden (23, 25). These methods are particularly relevant for airway image analysis because anatomical variability, scanner differences, and annotation inconsistencies frequently limit cross-institutional generalization. Broader self-supervised and domain-adaptation strategies remain relevant to medical imaging, but airway-specific evidence should be distinguished from results obtained on other anatomical targets.

F. Clinical airway analysis applications

While segmentation remains a fundamental component of airway image analysis, its clinical value lies in enabling reliable downstream measurements and representations. Automated airway–artery analysis, airway wall thickening and bronchiectasis quantification, airway tapering, central airway dimension assessment, and quantitative assessment of treatment-related airway change illustrate clinically relevant downstream uses (75–79). Airway labeling and graph-based anatomical representation can also support structured interpretation and navigation (44, 45, 81, 82). These applications demonstrate why topology and anatomical correctness matter beyond segmentation scores.

Nevertheless, technical capability should be separated from demonstrated clinical utility. Most computational airway studies are retrospective and many are evaluated on curated or internal datasets (36). External validation on independent institutions provides stronger evidence of robustness, but prospective clinical evaluation is required to establish workflow feasibility, and clinical-impact studies are needed to determine whether a system improves decisions, efficiency, or patient outcomes. Accordingly, this review treats segmentation and quantitative airway analysis as enabling technologies for future clinical applications rather than as evidence that clinical benefit has already been established.

G. Comparative analysis of method families

The evolution of airway image analysis does not establish a simple hierarchy in which each newer model family is universally superior. CNNs offer efficient local representation and stable training; transformers provide broader contextual modeling but generally increase computational cost; topology- and tubular-aware methods explicitly target connectivity, branch recovery, and structural plausibility; data-efficient strategies address annotation scarcity; and foundation models offer promising transfer and interactive capabilities but remain insufficiently validated for fine airway topology (6–11, 13–33). These trade-offs are more informative than chronological ranking.

Comparative evaluation should therefore consider at least volumetric accuracy, distal and branch-level completeness, topological correctness and leakage, computational requirements, annotation burden, and external generalization. A method that improves Dice on an internal dataset but fails to recover distal branches may be less useful for airway-tree analysis than a method with similar Dice and better BD or TD. Conversely, maximizing branch recovery at the cost of extensive leakage is not necessarily an improvement. Results obtained on different datasets should not be used to infer superiority without standardized head-to-head testing (36–38).

## 7 Discussion

A. Lessons learned from the evolution of airway image analysis

The literature reviewed in this work reveals an evolution from classical airway extraction toward deep-learning systems that increasingly model structural completeness and downstream quantitative use (1, 2, 4, 36, 37, 46, 47, 64–68, 73, 83, 84). This interpretation should be considered alongside the 2026 systematic review by Lsloum et al., which focused specifically on machine-learning methods for fully automated CT airway segmentation and found that the evidence base remains dominated by retrospective technical validation (36). The contribution of the present review is broader and systems-oriented: it links benchmark design, evaluation methodology, model families, annotation efficiency, foundation-model adaptation, quantitative airway analysis, and translational evidence. As segmentation performance improved, it became increasingly clear that overlap alone was insufficient for branching airway structures, motivating greater emphasis on connectivity, distal completeness, leakage, and anatomical plausibility (18–22, 37, 38, 74).

This realization has fundamentally influenced the development of modern airway image analysis systems. Recent research increasingly emphasizes topology preservation, branch completeness, structural plausibility, and clinically relevant quantitative assessment (18, 20–22, 44, 45). As a result, the field has gradually shifted from segmentation-centric methodologies toward broader airway analysis frameworks capable of supporting disease characterization, intervention planning, and quantitative airway assessment (44, 45, 53). The evolution of evaluation methodologies, network architectures, and benchmark datasets all reflect this transition.

B. Remaining challenges

Despite substantial progress, high-quality annotated airway datasets remain limited relative to the complexity of the task (36–39). ATM’22 represents an important advance in scale and diversity, while AeroPath introduces challenging pathology, but no single dataset captures the full range of scanner manufacturers, reconstruction protocols, respiratory phases, diseases, and clinical populations encountered in practice (38, 39). Manual annotation of distal airways remains technically demanding, and incomplete or heterogeneous ground truth can affect both training and apparent false-positive rates (21, 53). Future datasets should therefore document annotation extent, reviewer expertise, adjudication procedures, uncertainty, and quality-control rules.

Generalization across institutions remains problematic because airway appearance varies with scanner hardware, acquisition and reconstruction parameters, disease, respiratory phase, and population characteristics. Internal cross-validation is useful for model development but cannot establish robustness to new institutions. External validation should therefore be treated as a distinct level of evidence and should report the characteristics of both development and external cohorts (35, 36, 69–71). Domain adaptation and large-scale pretraining may reduce performance degradation, but they do not remove the need for independent testing. Distal airway reconstruction remains especially challenging in low-dose CT, pathology, and datasets with incomplete annotations (18–22, 48–53).

C. Emerging research directions

Several research trends are likely to shape the future development of airway image analysis systems. Among the most important is the growing adoption of topology-aware learning strategies that explicitly incorporate connectivity constraints, centerline information, graph representations, and anatomical priors into both model architectures and optimization procedures (18–22, 48, 74). These approaches directly address limitations associated with fragmented airway reconstructions and incomplete branch detection (18, 20–22).

Another important direction is the increasing use of foundation models and large-scale pretraining. Frameworks derived from Segment Anything, MedSAM, and related architectures demonstrate that knowledge acquired from large image collections can be successfully transferred to specialized medical imaging tasks. Such approaches may substantially reduce annotation requirements and improve generalization across datasets (29–33). Similarly, semi-supervised learning, weak supervision, active learning, and self-supervised representation learning offer promising solutions to the persistent scarcity of expert airway annotations (24–28).

At the same time, future research is expected to move beyond segmentation as an isolated task. Increasing emphasis is being placed on airway quantification, stenosis assessment, airway remodeling analysis, bronchial-tree characterization, intervention planning, and longitudinal disease monitoring (44, 45, 75–79, 81, 82, 85–87). These developments suggest that airway segmentation will increasingly serve as a foundational component within larger computational frameworks rather than as a standalone objective (88).

Explainability and interpretability are also relevant as airway image analysis systems move toward clinical decision support. *Post-hoc* approaches such as feature attribution and gradient-based visualization can help interrogate model predictions, while anatomically meaningful outputs—including airway centerlines, branch graphs, quantitative measurements, and intermediate prompt regions—may provide more directly interpretable information for airway applications (89–96). Related studies in clinical AI have explored explainable and interpretable approaches for prediction and decision support (97–99). However, explainability should complement rather than replace rigorous validation, and uncertainty estimation may provide additional information for identifying potentially unreliable predictions (100, 101).

D. Toward clinically deployable airway image analysis systems

The ultimate success of airway image analysis systems will depend on evidence that they can support real clinical workflows (35, 36). Improved segmentation accuracy alone is insufficient. Retrospective technical validation establishes algorithmic performance on a defined dataset; external validation tests generalization to independent data; prospective evaluation assesses feasibility within a real workflow; and demonstrated clinical utility requires evidence that use of the system improves a clinically meaningful process, measurement, decision, or outcome. These levels should not be conflated. Consequently, terms such as “clinically deployable” and “precision respiratory medicine” should be reserved for settings in which appropriate clinical evidence exists. Standardized benchmarks, multi-institutional validation, uncertainty reporting, and transparent failure analysis are necessary steps toward translation, but they are not themselves proof of clinical impact.

E. Key Takeaways for future airway image analysis systems

Several conclusions emerge from this review. First, methodological progress is increasingly driven not only by generic architectural innovation but by approaches tailored to airway structure, including connectivity-aware, topology-guided, tubular-feature, and sparse structural supervision (18–23, 48–53). Second, evaluation is moving beyond overlap toward branch, tree-length, centerline, and leakage measures that better characterize airway-tree quality (37, 38). Third, dataset diversity, annotation quality, and independent validation remain as important as model architecture (36, 38, 39, 70, 71). Finally, foundation models and data-efficient systems offer promising directions, but their airway-specific value must be established through direct structural validation rather than inferred from performance on other organs (23–33).

## 8 Conclusions

Airway image analysis has evolved from conventional image segmentation toward a broader computational framework encompassing anatomical reconstruction, topology preservation, quantitative assessment, and potential clinical decision support (1, 4, 5, 34–36, 73). This review examined the datasets, evaluation methodologies, and computational approaches that underpin modern airway image analysis systems, while distinguishing dedicated airway benchmarks from broader thoracic resources and general methodological foundations from airway-specific evidence. The reviewed literature demonstrates an evolution from CNN-based architectures toward transformer-enhanced, topology-aware, tubular-aware, data-efficient, and foundation-model-based approaches (6–11, 13–33, 48–53). However, this progression should not be interpreted as evidence that newer architectural families are universally superior.

Despite methodological advances, widespread clinical deployment remains insufficiently demonstrated. The scarcity of large, standardized airway datasets, variability in annotation quality, domain shifts across institutions, and difficulty of preserving distal airway connectivity remain important obstacles (36–39). Future systems should demonstrate independent multi-institutional robustness, reproducibility, clinically relevant quantitative performance, prospective workflow feasibility, and, where appropriate, measurable clinical impact. Segmentation performance, external validation, prospective evaluation, and clinical utility should be reported as distinct evidence levels.

The evolution of evaluation methodology is particularly important. Dice and related overlap metrics remain useful but cannot fully characterize a branching airway tree. Branch Detected, Tree Length Detected, centerline/connectivity measures, and leakage-related metrics provide complementary information about structural completeness and correctness (37, 38). Standardized reporting of these measures on shared benchmarks would substantially improve comparison across studies and reduce the risk of overinterpreting small gains in global overlap.

Looking forward, foundation-model adaptation, data-efficient learning, topology-preserving architectures, and multi-institutional benchmarking offer promising pathways toward more robust airway analysis (18–33, 38, 48–53). At the same time, future systems are likely to extend beyond segmentation toward quantitative airway assessment, disease characterization, intervention planning, longitudinal monitoring, and structured anatomical labeling (44, 45, 75–79, 81, 82, 85). These applications should be evaluated using downstream measurements and clinically relevant endpoints appropriate to the intended use rather than assuming that improved segmentation automatically produces clinical benefit.

Collectively, these developments provide a technical foundation for future quantitative respiratory assessment, image-guided intervention, and decision support. Progress toward those goals will depend not only on improved segmentation scores but on reliable datasets, anatomically meaningful evaluation, transparent validation, and evidence that computational performance translates into robust and clinically useful airway analysis (35, 36). Continued collaboration between engineers, computer scientists, radiologists, pulmonologists, and airway specialists will be essential for establishing that evidence.
